# Crystal Structure of ORF210 from *E. coli* O157:H1 Phage CBA120 (TSP1), a Putative Tailspike Protein

**DOI:** 10.1371/journal.pone.0093156

**Published:** 2014-03-26

**Authors:** Chen Chen, Patrick Bales, Julia Greenfield, Ryan D. Heselpoth, Daniel C. Nelson, Osnat Herzberg

**Affiliations:** 1 Institute for Bioscience and Biotechnology Research, University of Maryland, College Park, Maryland, United States of America; 2 Department of Chemistry and Biochemistry, University of Maryland, College Park, Maryland, United States of America; 3 Department of Veterinary Medicine, University of Maryland, College Park, Maryland, United States of America; Weizmann Institute of Science, Israel

## Abstract

Bacteriophage tailspike proteins act as primary receptors, often possessing endoglycosidase activity toward bacterial lipopolysaccharides or other exopolysaccharides, which enable phage absorption and subsequent DNA injection into the host. Phage CBA120, a contractile long-tailed Viunalikevirus phage infects the virulent *Escherichia coli* O157:H7. This phage encodes four putative tailspike proteins exhibiting little amino acid sequence identity, whose biological roles and substrate specificities are unknown. Here we focus on the first tailspike, TSP1, encoded by the orf210 gene. We have discovered that TSP1 is resistant to protease degradation, exhibits high thermal stability, but does not cleave the O157 antigen. An immune-dot blot has shown that TSP1 binds strongly to non-O157:H7 *E. coli* cells and more weakly to *K. pneumoniae* cells, but exhibits little binding to *E. coli* O157:H7 strains. To facilitate structure-function studies, we have determined the crystal structure of TSP1 to a resolution limit of 1.8 Å. Similar to other tailspikes proteins, TSP1 assembles into elongated homotrimers. The receptor binding region of each subunit adopts a right-handed parallel β helix, reminiscent yet not identical to several known tailspike structures. The structure of the N-terminal domain that binds to the virion particle has not been seen previously. Potential endoglycosidase catalytic sites at the three subunit interfaces contain two adjacent glutamic acids, unlike any catalytic machinery observed in other tailspikes. To identify potential sugar binding sites, the crystal structures of TSP1 in complexes with glucose, α-maltose, or α-lactose were determined. These structures revealed that each sugar binds in a different location and none of the environments appears consistent with an endoglycosidase catalytic site. Such sites may serve to bind sugar units of a yet to be identified bacterial exopolysaccharide.

## Introduction

During a bacteriophage infection cycle, binding of the virion to the host cell is achieved through a multi-step process called absorption whereby the phage first reversibly binds to a “primary” receptor and subsequently irreversibly binds a “secondary” receptor triggering release of phage DNA into the cell [1]. Phages with long tails (i.e. *Myoviridae* and *Siphoviridae*) accomplish binding to the primary and secondary receptors through various tail fibers. However, phages with short, non-contractile tails (i.e. *Podoviridae*) utilize tailspike proteins (TSPs) attached to the baseplate for binding of the primary receptor [2]. The primary receptor may be part of the core lipopolysaccharide (LPS), surface bound exopolysaccharides that extend beyond the LPS, or even carbohydrate components of the capsule. As such, the initial binding event is often located at some distance from the bacterial cell surface and degrading the polymer is essential for the phage to gain access to the outer membrane [3]. Significantly, all *Podoviridae* TSPs that have been studied in detail possess enzymatic activity against primary receptor polysaccharides. This enzymatic activity is essential for infection of environmental bacteria that are typically protected by a thick layer of long LPS.

*Podoviridae* TSPs are characterized by a 100–150 amino acid N-terminal head-binding domain that interfaces with the phage baseplate and a 400–600 amino acid C-terminal receptor binding domain that contains polysaccharide binding sites as well as an endoglycosidase catalytic site [1]. Amino acid sequence homology is readily identified between TSPs' head-binding domains whereas the receptor binding domains are notably divergent, often lacking any detectable sequence homology [4]. Crystal structures of a number of *Podoviridae* phage tailspikes have been determined including those from P22 [5], HK620 [6], SF6 [7], φ29 [8], and Det7 [9], and from the *Siphoviridae* phage 9NA [10]. These structures reveal that even in the absence of sequence homology, the receptor binding domains of the phages listed above are structurally related as all form homo-trimers and each subunit adopts primarily parallel right-handed 3-stranded β-helix folds [11]. The properties of this fold and its related members have been elegantly reviewed [12], [13]. The sugar-binding sites are located within the β-helix domain, either at the three interfaces between subunits or on the surfaces of each of the three subunits [1].

Bacteriophage CBA120 (vB_EcoM_CBA120), isolated from a cattle feedlot, was recently characterized against *Escherichia coli* and shown to infect 13 of 17 pathogenic strains bearing the O157:H7 serotype, but only 1 of 70 non-O157:H7 *E. coli* strains [14]. Further analysis of the CBA120 genome revealed it to be an unusual member of the *Myoviridae* family (i.e. phage with long, contractile tails, for example T4 phage). Specifically, it lacked all of the genes associated with outer baseplate proteins and the long tail fibers characteristic of *Myoviridae*. In contrast, CBA120 contained multiple genes for putative TSPs (TSP1-TSP4), genes that are more commonly associated with *Podoviridae* rather than *Myoviridae*. Comparative genomics of CBA120 and six, closely related, multi-tailspiked phages suggested they constituted a new genus within the *Myoviridae* family [15]. Thus, the “Viunalikevirus” genus, named for the phage ViI archetype, was established based on several distinguishing features including genome size and organization, gene synteny, use of a modified uracil instead of thymine, and the presence of four TSPs instead of the T4-like long tail fibers. Electron microscopy confirmed the presence of multiple star-like tailspike projections and an absence of long tail fibers in CBA120 as well as other members of the Viunalikevirus genus [15]. The four TSPs share amino acid sequence homology in the head-binding domain, but exhibit no detectable homology in the receptor-binding domain to one another or to any non-Viunalikevirus protein.

To better understand functional and structural diversity of tailspikes in general and the roles of the CBA120 tailspikes in facilitating host(s) infection, we have undertaken the characterization of the four CBA120 tailspike proteins. Here we show that the O157 antigen is not the receptor for TSP1, despite apparent specificity of the CBA120 phage for O157-bearing *E. coli* strains. We also show that similar to other TSPs, TSP1 is resistant to proteolysis and exhibits high thermal stability. We present the high resolution crystal structure of TSP1, and identify binding sites for three different sugars by X-ray crystallographic methods. TSP1 was submitted as a target for structure prediction prior to structure determination during CASP10 community experiment [16]. A brief structure description and predictions' evaluation from the experimentalist viewpoint have been published [17].

## Materials and Methods

### Cloning, Expression, and Purification

The nucleic acid sequence of open reading frame 210 (i.e. *tsp1*) of the CBA120 phage genome (accession no. NC_016570.1) was codon-optimized for expression in *E. coli*, commercially synthesized by GeneArt (Regensburg, Germany) including a C-terminal 6X-His tag, sub-cloned into the pBAD24 plasmid [18], and transformed into BL21 cells. For expression, cells were grown in Luria-Bertani (LB) broth supplemented with 100 µg/mL ampicillin at 37°C for 4 hours followed by induction with 0.25% arabinose for an additional 4 hours. TSP1 was purified following cell lysis via sonication and centrifugation at 13,000 rpm for 1 hr by an IMAC Profinity column (Bio-Rad). TSP1 was then dialyzed in PBS, pH 8, followed by gel filtration using an S-200 column (GE Healthcare) to achieve homogeneous purity.

To make a selenomethionine derivative of TSP1, pBAD24::*tsp1* was transformed into B834 cells (Novagen) and methionine auxotrophy was confirmed by plating on SelenoMet™ media (Molecular Dimensions) with or without supplemental methionine. Selenomethionine-TSP1 was produced using similar conditions as wild-type but with SelenoMet™ media and 40 mg/L selenomethionine. Purification protocol was identical to that used for the wild-type protein.

### Analytical Gel Filtration

Analytical gel filtration was used to determine the multimeric state of native TSP1. A total of 100 µL (100 µg) of TSP1 was applied to a pre-equilibrated Superose 6 gel filtration column (GE Healthcare) and run under isocratic conditions in PBS for 1.5 column volumes on an AKTA FPLC system (GE Healthcare). Molecular mass of TSP1 was estimated from a standard curve (linear regression of log(molecular mass) against retention volume) generated using gel filtration standards (Bio-Rad).

### LPS Glycosidase Assay

To test TSP1 for LPS glycosidase activity, *E. coli* O157 LPS was extracted from ATCC strains 43894 and 700728 according to the phenol-water method of Westphal [19] as modified by Rezania [20]. Alternatively, O157 LPS was purchased from List Biological Laboratories. *E. coli* O157 LPS (1.5, 15, or 75 µg) was incubated with 1.5, 7.5, or 15 µg TSP1 overnight at 37°C, subjected to SDS-PAGE, and silver stained to observe evidence of LPS degradation.

### TSP1 Binding Assays

A dot blot was used to evaluate binding of TSP1 to the bacterial surface. *E. coli* O157:H7 strains (ATCC 43894 and 700728), non-O157 *E. coli* strains (ATCC 35218, DH5α), and *Klebsiella pneumonia* (ATCC 700603) were grown overnight and 4 µL of each were spotted on a nitrocellulose membrane (Ambion). In addition, 5 µg of His-tagged TSP1, PlyC (an unrelated protein control), or His-tagged PlyC were spotted as positive and negative controls for that antibody detection. Fresh 10 mL aliquots of a solution containing 20 mM phosphate buffer, pH 7.0, supplemented with 0.1% (v/v) Tween 20 and 3% bovine serum albumin were used for all blocking and washing steps. The membrane was sequentially washed and incubated in 1 hr intervals with purified His-tagged TSP1 (100 µg/mL), a 1∶1000 dilution of mouse anti-His primary antibody (GenScript), and a 1∶1000 dilution of a goat anti-mouse IgG (HRP) secondary antibody conjugated to horse radish peroxidase (GenScript). The signal indicating binding was detected using the SuperSignal™ West Pico Chemiluminescent Substrate kit (Thermo Scientific).

In an alternative cell binding assay, TSP1 (8 mg) was fluorescently labeled by crosslinking to AlexaFluor 555 (Molecular Probes) via primary amines through a tetrafluorophenyl ester according to the product instructions. The reaction was quenched by addition of 100 mM Tris and fluorescent TSP1 was desalted to remove unreacted dye. Labeled TSP1 (10–100 µg) was mixed with 0.5 ml of an overnight culture of *E. coli* O157:H7 (ATCC 43894 and 700728) resuspended in PBS. The cells were further washed twice in PBS and viewed by fluorescent microscopy (Nikon Eclipse 80i) to elucidate binding of TSP1.

### Thermal Stability by Circular Dichroism (CD) Spectropolarimetry

CD experiments were performed on a Chirascan CD Spectrometer (Applied Photophysics) equipped with a thermoelectrically controlled cell holder. For melting experiments, TSP1 at a 0.1 mg/mL concentration in 20 mM sodium phosphate buffer, pH 7, was heated from 20°C to 95°C using a 1°C/min heating rate. The mean residue ellipticity (MRE) was monitored at 218 nm in a 1 mm path length quartz cuvette at 0.5°C steps with 5 second signal averaging per data point. The resulting melting data were smoothed, normalized, and fit with a Boltzmann sigmoidal curve using the Pro-Data software (Applied Photophysics). The first derivative of the melting curve was taken to determine the melting temperature (T_m_) of the sample, which was defined as the minimum in the derivative graph.

### Susceptibility to SDS and Proteolysis

Sensitivity to SDS was determined by incubating purified TSP1 at a 0.25 mg/mL concentration in Laemmli Sample Buffer (Bio-Rad) (1% final SDS concentration) for ten minutes at room temperature or 100°C followed by qualitative analysis on a 7.5% SDS-PAGE gel. To analyze enzyme vulnerability to proteolysis, TSP1 was incubated at a concentration of 0.5 mg/mL with either trypsin (Thermo Fisher Scientific) or chymotrypsin (Sigma-Aldrich) at a 1∶25 (w/w) protease∶TSP1 ratio in 20 mM sodium phosphate buffer, pH 7, containing 1 mM CaCl_2_ at 37°C overnight. Samples were then investigated for proteolytic degradation by SDS-PAGE. Bovine serum albumin (BSA) (New England Biolabs) served as a control in both experiments.

### Crystallization and Structure Determination

Both, wild-type and seleno-methionine (Se-Met) containing TSP1 crystals were obtained by the vapor diffusion method in hanging drops at room temperature, with the reservoir solution containing 0.1 M Tris-HCl (pH 7.0–7.6), and 16% w/v polyethylene glycol 1000. Large crystals of approximately 0.2×0.2×0.4 mm^3^ appeared within a couple of days. The crystals were transferred into mother liquor supplemented with 10% glycerol and then flashed cooled in liquid nitrogen. X-ray diffraction data were collected on the synchrotron beamline 23-ID, General Medical Sciences and National Cancer Institute Collaboration Access Team (GM/CA-CAT), at the Advanced Photon Source, Argonne National Laboratory (Table 1). The beamline was equipped with a MARmosaic MX-300 detector (Marresearch). A Se-Met protein crystal was used to determine the structure by MAD methods, exploiting the Se absorption edge and collecting a 3-wavelength dataset to the resolution limit of 2.2 Å. In addition, 1.8 Å resolution datasets were collected for refinement of the Se-Met and wild-type TSP1 structures, and a 2 Å resolution wild-type TSP1 dataset was collected at the Zn absorption edge peak to verify the presence of Zn^2+^ bound to the N-terminal domain. To identify potential sugar binding sites, wild-type TSP1 crystals were briefly soaked in cryo-protected mother liquor containing 37% saturated glucose, mannose, α-lactose, or α-maltose. Diffraction data were collected using the in-house X-ray facility consisting of a Rigaku MicroMax 007HF rotating anode generator (CuKα radiation) and a RAXIS IV^++^ imaging plate detector (Table 2). All datasets were processed with the computer program XDS [21]. Data processing statistics are provided in Tables 1 and 2. Structure factors were calculated using the program TRUNCATE [22] as implemented in CCP4 [23]. 5% randomly selected reflections were set aside for calculation of the free-*R* values [24].

**Table 1:** Statistics on data collection, phasing, and refinements of CBA120 TSP1 structure.

**Data Collection**					
	Se-Met TSP1				Wild-type TSP1
	Peak^b^	Inflection^b^	High Remote^b^	Low Remote^c^	
Wavelength (Å)	0.97945	0.97961	0.94945	1.03320	1.03320
Space Group	P2_1_2_1_2_1_				
Cell Dimension (Å)	123.23, 153.09, 171.44				123.61, 147.82, 170.63
aResolution (Å)	20–2.2 (2.26–2.2)	20–2.2 (2.26–2.2)	20–2.2 (2.26–2.2)	90–1.8 (1.85–1.8)	73.9–1.8 (1.85–1.8)
aCompleteness (%)	99.0 (96.3)	98.7 (95.1)	98.9 (95.7)	99.6 (99.1)	99.8(99.6)
No. of unique Reflections	316917	317685	317478	297461	298512
aRedundancy	3.4 (2.6)	3.4 (2.6)	3.4 (2.6)	6.5 (4.2)	6.7 (4.9)
a *R*_merge_	0.087 (0.363)	0.096 (0.448)	0.095 (0.413)	0.087 (0.500)	0.094 (0.510)
a *I*/σ(*I*)	10.3 (2.6)	9.4 (2.0)	9.6 (2.2)	13.3 (2.1)	11.9 (2.2)
**MAD-Phasing**					
aResolution	20–2.3 (2.41–2.30)				
No. of Se atoms (found/correct)	32/29				
aInitial figure of merit (FOM)	0.46 (0.27)				
aFOM after density modification	0.67 (0.32)				
**Refinement**					
	Se-Met TSP1				Wild-type TSP1
aResolution (Å)	90–1.8 (1.82–1.8)				63.4–1.8 (1.85–1.8)
a *R*_work_	0.187 (0.369)				0.189 (0.375)
a *R*_free_	0.204 (0.384)				0.207 (0.398)
RMSD bond length (Å)/bond angle (°)	0.008/1.1				0.007/1.4
Ramachandran plot: Favored/Allowed/Disallowed (%)	86.6/13.2/0.2				87.0/12.9/0.1
No. of protein atoms	16865				16972
No. of Zn^2+^ ion	1				1
No. of water molecules	2361				2449

a The values in parentheses are for the highest resolution shell.

*R*_merge_ = Σ*_hkl_* [(Σ*_j_*|*I_j_*−<*I*>|)/Σ*_j_*|*I_j_*|].

*R*_work_ = Σ*_hkl_*| |*F_o_*|−|*F_c_*| |/Σ*_hkl_*|*F_o_*|, where *F_o_* and *F_c_* are the observed and calculated structure factors, respectively.

*R*_free_ is computed from 5% randomly selected reflections that were omitted from the refinement.

b data processed with anomalous signal.

c data processed without anomalous signal (for refinement only).

**Table 2:** Statistics on data collection and refinements of sugar-bound TSP1 CBA120 structures.

**Data Collection**			
Wavelength (Å)	1.5418		
Ligands	TSP1/Glucose	TSP1/Lactose	TSP1/Maltose
aResolution (Å)	43.8–2.0 (2.05–2.0)	76–2.0 (2.05–2.0)	61.7–1.95 (2.0–1.95)
aCompleteness (%)	96.2 (92.2)	97.0(93.3)	98.5(88.0)
No. of unique Reflections	209831	211941	224635
aRedundancy	6.3 (4.7)	3.5(2.7)	5.9(3.3)
a *R*_merge_	0.107 (0.439)	0.082(0.482)	0.107(0.475)
a *I*/σ(*I*)	13.0 (2.9)	11.3(2.2)	12.0(2.0)
**Refinement**			
aResolution (Å)	43.8–2.0 (2.02–2.0)	76.0–2.0 (2.05–2.0)	61.7–1.95 (2.0–1.95)
a *R*_work_	0.177 (0.293)	0.177 (0.269)	0.189 (0.359)
a *R*_free_	0.209 (0.313)	0.210 (0.294)	0.218 (0.381)
RMSD bond length (Å)/bond angle (°)	0.007/1.5	0.007/1.4	0.008/1.4
Ramachandran plot: Favored/Allowed/Disallowed (%)	86.6/13.3/0.1	86.8/12.6/0.5/0.1	87.1/12.2/0.6/0.1
No. of protein atoms	17005	17005	16988
No. of Zn^2+^ ion	1	1	1
No. of ligand molecules	4	3	3
No. of water molecules	2372	2275	2512

a The values in parentheses are for the highest resolution shell.

*R*_merge_ = Σ*_hkl_* [(Σ*_j_*|*I_j_*−<*I*>|)/Σ*_j_*|*I_j_*|].

*R*_work_ = Σ*_hkl_* | |*F_o_*|−|*F_c_*| |/Σ*_hkl_* |*F_o_*|, where *F_o_* and *F_c_* are the observed and calculated structure factors, respectively.

*R*_free_ is computed from 5% (TSP1/Glucose) or 2,000 (TSP1/Lactose and TSP1/Maltose) randomly selected reflections that were omitted from the refinement.

The phases were determined by the MAD method with the software PHENIX AutoSol [25], which incorporates the programs Hyss for heavy atom search, SOLVE for phasing, and RESOLVE for density modification. 29 Se atoms were identified, yielding an initial overall figure of merit of 0.46 at the resolution limit of 2.3 Å. Density modification calculated with the program RESOLVE [26], including 3-fold non-crystallographic symmetry averaging, improved the overall figure of merit to 0.67 (Table 1). The quality of the resulting electron density map was excellent, allowing the automated model building program Arp/wArp [27] to trace a nearly complete TSP1 polypeptide chain. Subsequent cycles of manual model rebuilding were performed with the interactive computer graphics program XTALVIEW [28]. Structure refinements were carried out with CNS [29] and PHENIX [30].

The refined Se-Met protein structure was used as the initial model for structure determinations of the wild-type TSP1 as well as the three TSP1/sugar complexes. Water molecules were automatically built using the program PHENIX. Towards the end of the refinement, the models of the glucose, α-lactose, and maltose molecules were fitted in the respective electron density maps. The quality of each structure was validated with the program PROCHECK [31]. The location of Zn^2+^ was determined from the anomalous differences collected at the peak wavelength of the zinc absorption edge.

The embedded surface area was calculated with AREAIMOL as implemented in CCP4 [23]. The figures were prepared with the program PYMOL [32]. The coordinates and structure factors were deposited in the Protein Data Bank (entry codes 4OJ5 for wild-type TSP1, 4OJ6 for SeMet TSP1, 4OJL for TSP1/glucose complex, 4OJO for TSP1/lactose complex, and 4OJP for TSP1/maltose complex.

## Results and Discussion

### TSP1 Biochemical and Biophysical Properties

**Figure 1:**
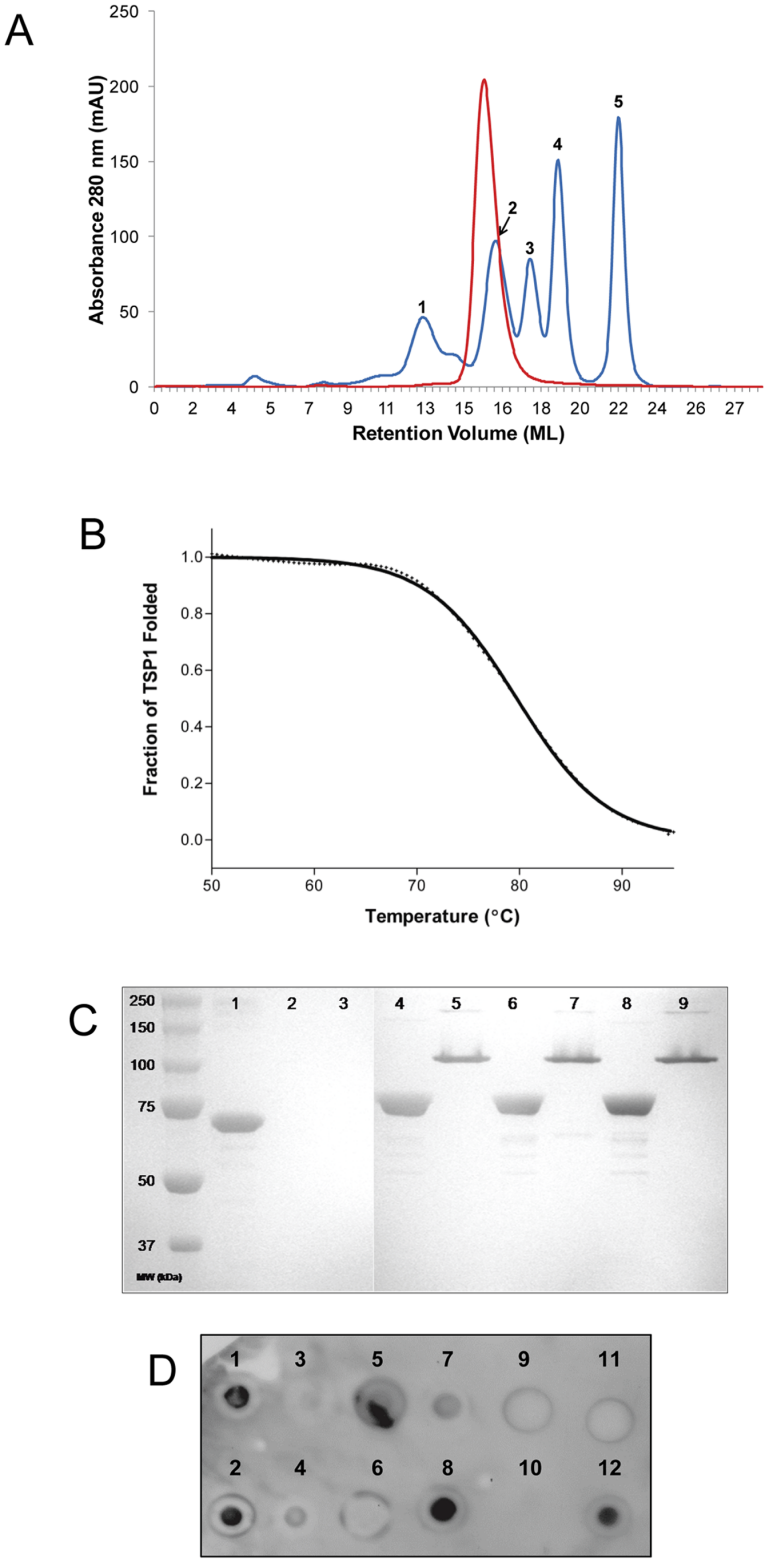
Biophysical/biochemical characterization of TSP1. (A) Analytical gel filtration of purified TSP1 (red). Predicted monomer is 82 kDa and a trimer is 246 kDa. Gel filtration standards (blue) are as follows: (1) Thyroglobulin, 670 kDa (void volume); (2) Gamma globulin, 158 kDa; (3) Ovalbumin, 44 kDa; (4) Myoglobin, 17 kDa; (5) Vitamin B_12_, 1.35 kDa. (B) Thermal unfolding monitored by CD spectroscopy. The experimental values are indicated by+symbols and the continuous line corresponds to the theoretical curve. (C) TSP1 SDS and protease stability. Lanes: (1) BSA; (2) BSA+trypsin; (2) BSA+chymotrypsin; (4) TSP1 boiled; (5) TSP1 non-boiled; (6) TSP1+trypsin, boiled; (7) TSP1+trypsin, non-boiled; (8–9) As with 6–7 but with chymotrypsin. (D) Immuno-dot blot. (1) *E. coli* ATCC 35218 (Non-O157:H7); (2) *E. coli* DH5α (Non-O157:H7); (3) *E. coli* ATCC 700728 (O157:H7); (4) *E. coli* ATCC 43894 (O157:H7); (5) O157 LPS from ATCC 43894; (6) O157 LPS from List Biologicals; (7) *K. pneumoniae* ATCC 700603; (8) His-tagged TSP1 (positive control); (9) Buffer (negative control); (10) empty; (11) PlyCB (negative control protein); (12) His-tagged PlyCB (positive control protein).

Both the wild-type and selenomethionine derivative of TSP1 yielded several milligrams of soluble protein per liter of bacterial culture. Initially, a TSP1 construct containing an N-terminal 6X-His tag failed to bind the nickel IMAC column unless denatured with urea, indicating that the N-terminus was not solvent accessible. Nonetheless, a construct containing a C-terminal 6X-His tag did bind the IMAC column and was used for all subsequent experiments. Analytical gel filtration of purified TSP1 revealed a single homogeneous peak (Figure 1A) at ∼252 kDa based on regression analysis of gel filtration standards (data not shown), suggesting that similar to all other tailspike proteins with known structures [1], TSP1 forms a trimer in solution (predicted 82 kDa monomer, 246 kDa trimer).

In addition to formation of a trimer, tailspike proteins that have been studied in detail are usually characterized by high thermal stability, tolerance of SDS, and resistance to proteolytic degradation. To analyze the thermal stability of TSP1, the loss in β-sheet content from 20°C–95°C was monitored at 218 nm by CD spectroscopy. The resulting TSP1 melting curve displays an uncooperative unfolding transition that correlates to a T_m_ of 80.7°C (Figure 1B). These results are consistent with those found for the P22 tailspike (T_m_ = 88.4°C) [33] and the HK620 tailspike (T_m_ = 80°C) [6].

Next, the structural integrity of TSP1 was investigated when subjected to either anionic detergent or protease treatment. In the presence of SDS, TSP1 remained folded in a non-denatured state (Figure 1C, lane 5). Although the band on the SDS-PAGE does not correlate to the ∼246 kDa trimer, it is well documented that the mobility of non-denatured multimers is greater than the mass would predict and this phenomenon is seen with other tailspikes under similar conditions [6]. In contrast, when SDS-treated TSP1 sample was boiled for several minutes, a completely denatured soluble monomer at ∼82 kDa was noted (Figure 1C, lane 4). As a control, BSA (66 kDa) was denatured when introduced to SDS only (Figure 1C, lane 1). To assess the proteolytic susceptibility of TSP1, the tail spike protein was incubated with either trypsin or chymotrypsin overnight at 37°C. Neither of the two proteases had any effect on the structural integrity of TSP1, as evident by the absence of TSP1 degradation (Figure 1C, lanes 6–9) when compared to the TSP1 samples without protease treatment (Figure 1C, lanes 4–5). To assure both proteases were catalytically active, trypsin and chymotrypsin were incubated with BSA using the same buffer and incubation conditions as the TSP1 experiment. Both trypsin and chymotrypsin effectively degraded BSA, resulting in no observable BSA protein following proteolytic degradation (Figure 1C, lanes 2–3).

### Overall Crystal Structure

**Figure 2:**
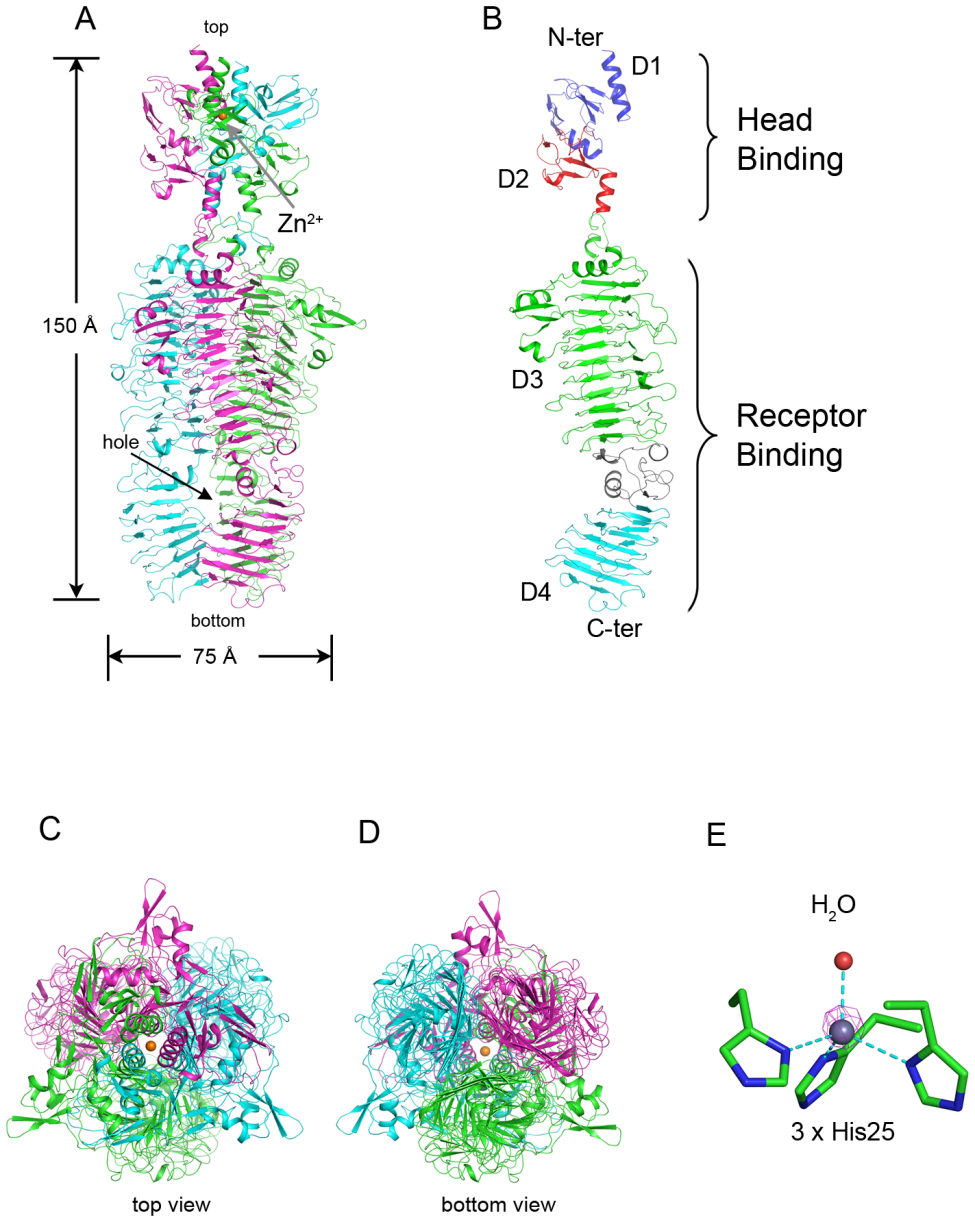
Structure of TSP1 from bacteriophage CBA120. (A) “Side view” of the homotrimer. The three monomers are colored in green, cyan, and magenta. The Zn^2+^ is shown as an orange sphere and indicated by an arrow. The “hole” in the catalytic domain is indicated. (B) The structure of TSP1 monomer. The N-terminal head binding domain and a C-terminal receptor-binding domain are further divided into four subdomains, D1, D2, D3, and D4 colored in blue, red, green, and cyan, respectively. The D3-D4 intervening region that bends the β-helical axis is colored in grey. (C) and (D) “Top view” (down from the N-terminus) and “bottom view” (down from the C-terminus), respectively. (E) Anomalous difference map calculated with diffraction data collected at the zinc absorption edge peak (1.28283 Å). The calculated phases included only the protein atoms. The Zn^2+^ coordinates His25 of each subunit and a water molecule. The anomalous difference map (magenta cage) is contoured at 15σ.

The full-length CBA120 TSP1 contains the encoded 770 amino acid residues followed by six histidine residues at the C-terminus, which were added for protein affinity purification. The crystal's asymmetric unit contains the biological homotrimer (Figure 2). The first 10–14 N-terminal residues, the last 1–2 residues of the three subunits, and the His-tag were not resolved in the electron density maps and therefore were not modeled. The root mean square deviation (rmsd) values between the subunits are 0.9 Å for all backbone atoms. A Zn^2+^, verified by the anomalous diffraction at the zinc absorption edge, forms tetrahedral coordination with three His25 imidazole groups, each located on the N-terminal α-helices of a TSP1 subunit and with a single water molecule (Figure 2E).

The TSP1 trimer assumes an elongated rod-like shape of approximately 170 Å in length and 75 Å in diameter at the widest region (Figure 2A). The three subunit interfaces embed a total of 22,000 Å^2^ surface area, burying nearly a quarter of the 31,000 Å^2^ of each subunit surface.

TSP1 monomer contains two functional domains (Figure 2A). The N-terminal domain (amino acid residues 12–155) that putatively attaches to the virion forms the spherical head of the trimeric assembly (Figure 2A). The C-terminal domain (amino acid residues 166–769) forms a bent 3-stranded right-handed parallel β helix. By analogy to other tailspike proteins, this is the putative receptor binding domain that binds and hydrolyzes the bacterial LPS [1]. Together, the three receptor binding domains of the TSP1 homotrimer form a left-handed coiled β-coil structure (Figure 2A), resembling other tailspike proteins of known structures. A short α-helix (amino acid residues 155–165) forms a “neck” that connects the head binding and receptor binding domains. The neck has been seen previously in tailspike protein structures including those produced by recombinant DNA techniques without their head binding domains.

**Figure 3:**
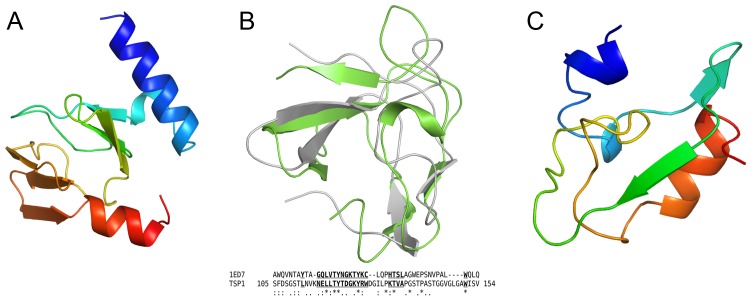
Folds of non β-helix subdomains of TSP1. (A) Overall fold of the D1 subdomain. The chain is colored progressively from blue (N-terminus) to red (C-terminus). (B) Structure homology between subdomain D2 (green) and the chitin binding domain of Chitinase from *Bacillus circulans* (gray). The structure based sequence alignment shows the well-superposed residues in bold underlined letters. Invariant residues are marked by *. (C) Overall fold of the D3-D4 linker region. The chain is colored progressively from blue (N-terminus) to red (C-terminus).

The TSP1 head binding domain can be further divided into two subdomains (Figure 2B), each beginning with a α-helix followed by an anti-parallel β-sandwich, D1 (residues 12–96) and D2 (residues 97–154). In addition to electrostatic and hydrophobic interactions, oligomerization of the three head binding domains is enhanced by the coordination to the Zn^2+^. A Dali structure homology search [34] revealed no significant structure analogs of the subdomain D1, thus the β-strand topology of this β-sandwich is novel (Figure 3A). In contrast, the Dali search revealed that subdomain D2 folds similarly to the NMR structure of the chitin binding domain of Chitinase from *Bacillus circulans* ([35], PDB entry code 1ED7) with rmsd of 2.1 Å over 38 paired Cα atoms and very low amino acid sequence identity (Figure 3B). The significance of the structural homology to the chitin binding domain is unknown as binding of tailspike head binding domains to polysaccharides has never been reported.

The receptor binding domain may be further divided into two β-helical subdomains D3 (residue 166–562) and D4 (residues 624–769), intervened by a non β-helical region (residues 563–623). Both D3 and D4 adopt a right-handed β-helix fold consisting of 3 β-stranded coil turns. D3 begins with an α-helix that caps the β-helix as seen in other tailspike protein structures. The D3-D4 intervening region breaks the β-helix. A Dali search did not reveal structural homologs of this region (Figure 3C). The beginning of this region follows the subdomain D3 coiling trajectory but introduces two 1-turn helices instead of β conformations. These are followed by two β-strands. The first β-strand stacks against the last β-strand of subdomain D3 to extend one of its β-sheets and the second β-strand stacks against the first β-strand of subdomain D4. Next, the polypeptide chain meanders in the reverse direction of the β-helix axis and ends with a 3-turn α-helix, after which the coiling direction is resumed. The inserted region introduces a 30° bend between the D3 and D4 β-helix axes (Figure 2B). This bending produces a 13 by 16 Å channel along the trimer axis with openings to bulk solvent between each of the trimer subunits. In contrast, extensive contacts are observed between subunits along all other subdomains (Figure 2B). Two of the three sugars soaked into the crystals bind in this “hole”, as described below.

The D3 β-helix contains 11 turns and the D4 β-helix contains 7 turns, with some edge turns exhibiting perturb β-strands. Contacts in the center of the β-helices are governed by hydrophobic interactions, whereas intermolecular contacts between β-helices are predominantly hydrophilic.

**Figure 4:**
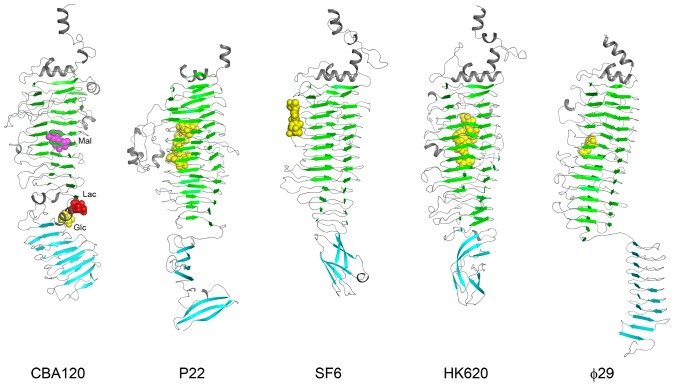
Structures of the receptor binding domains of TSP1 and of other tailspike proteins. All molecules are shown in the same orientation after alignment with subdomain D3 using Dali. The β-strands of subdomains D3 and D4 are highlighted in blue and cyan, respectively. Locations of bound sugars are indicated in space filling models. PDB entries for the molecules displayed are as follows: CBA120 – 4OJL, 4OJO, 4OJP; P22 - 1TYX; SF6 - 2VBM; HK620 - 2VJJ; φ29 - 3GQ8.

For the TSP1 receptor binding domain, the closest hits from a Dali search were β-helices of other phage tailspike proteins (Figure 4) including those from phages P22 ([5], PDB code: 1TYX), HK620 ([6], PDB code: 2VJJ), SF6 ([7], PDB code: 2VBM), and φ29 ([8], PDB code: 3GQ8). Despite the similarity of the fold, these proteins lack amino acid sequence homology to TSP1. Superposition of subdomain D3 β-helix with the various tailspike proteins yielded paired Cα atom r.m.s.d. values in the range of 3 Å, whereas the loops connecting the β-strands are strikingly dissimilar. TSP1 subdomain D4, which also adopts a right-handed 3-stranded β-helical fold, exhibits no structure homology to receptor-binding C-terminal subdomains of other tailspike proteins, although the latter adopt various β structures. Even the β-helix seen in C-terminal domain of φ29 tailspike [8] is very different because it contains 2 β-stranded coils, termed β-rolls in the nomenclature of Yoder and Jurnak [13], and its helical axis is cyclically swapped around the 3-fold trimer axis compared with the disposition of the N-terminal receptor binding subdomains (Figure 4).

### Sugar Binding Sites

**Figure 5:**
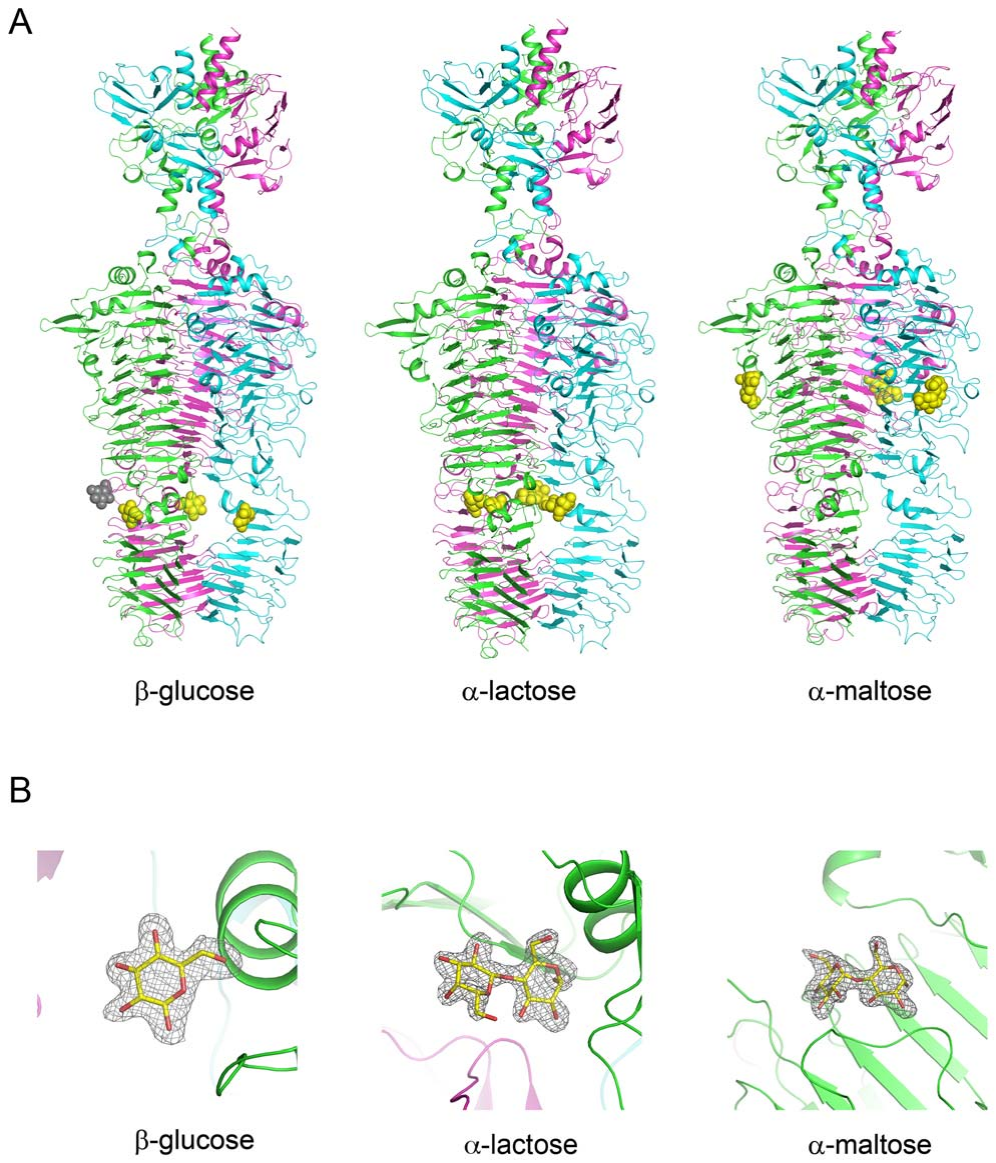
TSP1 sugar binding sites revealed by crystallographic soaking experiments. (A) The sugar binding sites shown in the context of the overall structure of TSP1. The cartoon colors of monomers are as in Figure 2. The sugars are shown as space filling models and colored in yellow, except for a fourth glucose molecule colored gray, depicting a glucose involved in crystal contacts. (B) The σA weighted *2F*o-*F*c electron density maps associated with the sugars, omitting the sugar from the calculated structure factors. The maps are contoured at 1σ level. The glucose is in the β-conformation, and the lactose and maltose are in an α-conformation.

Although the substrate of TSP1 from CBA120 bacteriophage remains unknown (see below), the presence of a β-helix domain analogous to other tailspike proteins suggests that the protein binds polysaccharides. To identify possible sugar binding sites, the crystals were flash soaked in high concentrations of readily available mono-saccharides (mannose and glucose) and di-saccharides (α-lactose and α-maltose). Of these, binding sites of glucose, α-lactose, and maltose were evident in the difference electron density maps. Each sugar binds at the same site on each of the TSP1 trimer subunits. However, the different sugars bind at different locations (Figures 4 & 5).

The TSP1/glucose complex revealed a fourth molecule (colored gray in Figure 5) bound in a niche generated by crystal packing between two crystallographically related monomers (chain C in the coordinate set deposited in the PDB). Because of the involvement of crystal contacts, this fourth site might not be physiologically relevant; hence only the interactions of the three equivalent glucose molecules that are independent of crystal contacts are discussed below.

**Figure 6:**
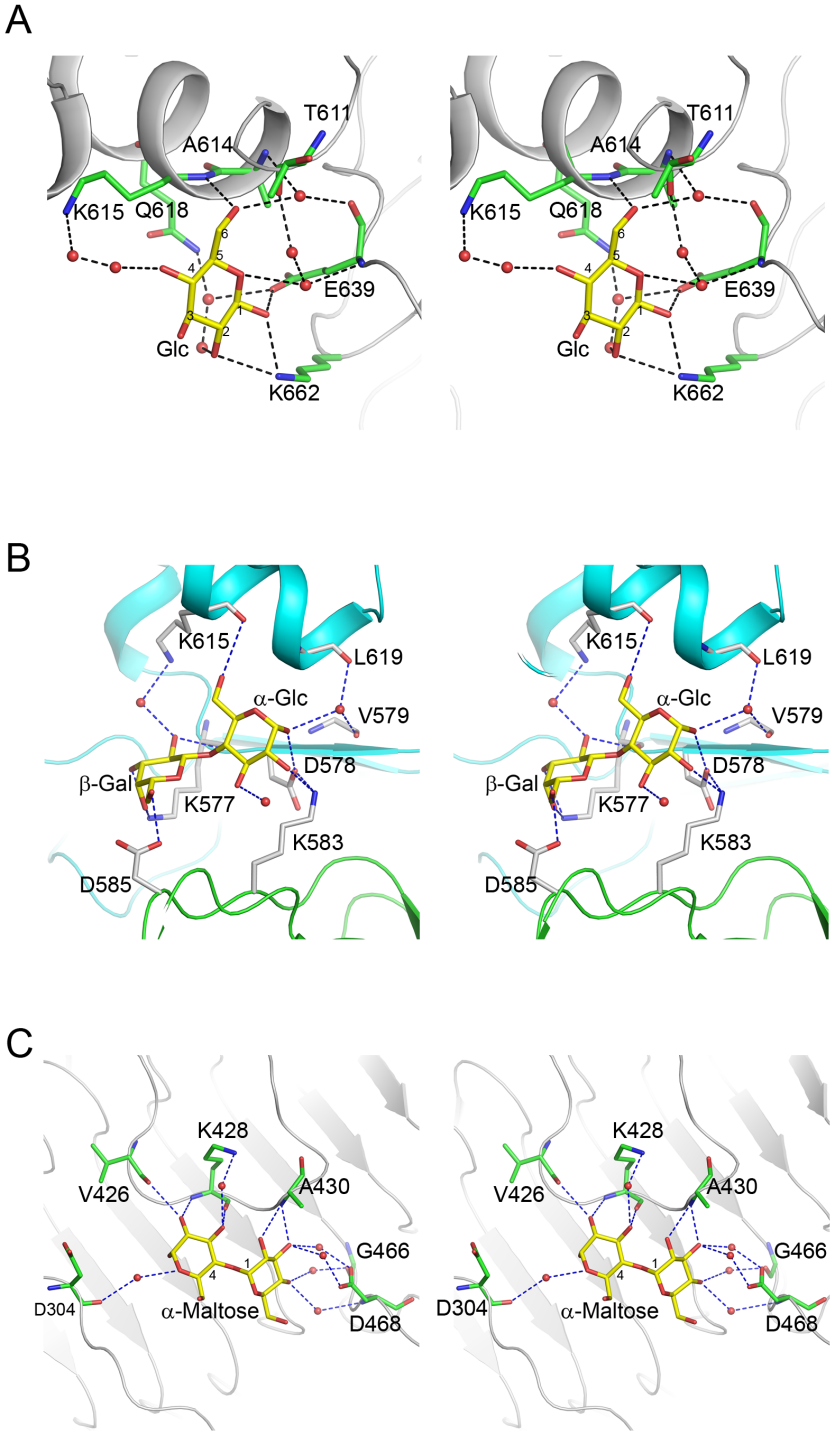
Stereoscopic representation of the environment of sugars bound to TSP1. Ligands and interacting protein residues are shown as stick models and water molecules as spheres. The atom color scheme is as follows: Nitrogen – blue, oxygen – red, ligand carbon – yellow, protein carbon – green. Hydrogen bonds are shown as dashed lines. (A) Glucose. (B) Lactose. (C) Maltose. The cartoon models are colored gray in (A) and (C). The cartoon models of two TSP1 molecules that contribute to the lactose binding are colored green and cyan in (B).

All glucose molecules exhibit the β conformation. The binding site is located at the periphery of the hole generated by the D3-D4 intervening region, adjacent to subdomain D4 (Figures 4 & 5). The glucose engages a single subunit and the interactions include both direct hydrogen bonds to the protein ligand-water-protein bridged hydrogen bonds (Figure 6A): The carboxylate group of Glu639 forms a hydrogen bond with the C1 hydroxyl group. The amine group of Lys662 forms hydrogen bonds with both the C1 and C2 hydroxyl groups. The backbone amide of Lys615 is hydrogen bonded to the primary alcohol hydroxyl group of C6. A water mediated interaction bridges the backbone carbonyl of Glu639 and the C6 hydroxyl group. A second water molecule bridges the backbone amide of Glu639 and the hemiacetal oxygen atom of the glucose.

The α-lactose also binds in the hole formed by the D3-D4 intervening region, but it inserts more deeply into the hole compared with the glucose and interacts with two subunits. Again, hydrogen bond interactions include both the protein backbone and side chains and bridging water molecules. In contrast to the interactions of the β-glucose, which are identical for all three molecules, the interactions of the three α-lactose molecules with the protein are similar but not identical which is manifested in the pliability of some side chain conformations. Figure 6B shows one example of the binding mode. The invariant interactions seen in in all three binding sites are as follows: For the α-glucose moiety, the carbonyl oxygen atom of Val579 backbone forms a hydrogen bond with a water molecule, which is in turn hydrogen bonded to C1. For the β-galactose moiety, the backbone carbonyl oxygen of Lys577 interacts with the C2 hydroxyl group concomitantly with the side chain amine group forming hydrogen bonds with both C3 and C4 hydroxyl groups. The carboxylate group of Asp585 on a neighboring TSP1 subunit interacts with the C6 hydroxyl group.

The α-maltose binds intramolecularly on the surface of subdomain D3 of the receptor binding domain in a shallow depression (Figure 6C). The disaccharide stacks above a cluster of three aromatic side chains, Tyr306, Tyr427, Phe443. Direct interactions between the non-reducing glucose moiety and the protein consist of the Ala430 backbone amide group forming a bifurcated hydrogen bond with the C2 and C3 hydroxyl groups. Water molecules bridged hydrogen bonds include Asp468 carboxylate group with the C3 and C4 hydroxyl groups, and the backbone amide group of Asp468 with the C4 hydroxyl group. In the reducing glucose moiety, direct sugar protein interactions include those of the C2 hydroxyl group with the backbone carbonyl oxygen of Val426 and the backbone amide of Lys428, and the C3 hydroxyl group with the backbone carbonyl oxygen of Lys428. The C3 hydroxyl group is also bridged by a water molecule to the side chain amine of Lys428. In two out of the three maltose molecules, the hemiacetal oxygen and the backbone carbonyl oxygen of Asp304 are bridged by a water molecule.

### What is the substrate for TSP1?

Despite LPS hydrolysis activity noted with most phage tailspike proteins and confirmation of various sugar binding sites on TSP1 by X-ray crystallography, we found no evidence of *E. coli* O157 LPS hydrolysis by TSP1 on extracted LPS from two different strains (ATCC 43894 and 700728) or LPS purchased from a commercial vendor (data not shown). Thus, the substrate for TSP1 remains unknown. Presumably one of the other three TSPs of the CBA120 phage is responsible for this activity. Moreover, receptors other than LPS have been identified for some tailspike proteins [1]. To test for this possibility, we performed an immune-dot blot assay to elucidate potential binding of TSP1 to any epitope on the bacterial surface (Figure 1D). Much to our surprise, TSP1 displayed strong binding to non-O157:H7 *E. coli* cells (spots 1, 2) and even weak binding to *K. pneumoniae* cells (spot 7), but little to no detectable binding to *E. coli* O157:H7 strains (spots 3, 4) despite the apparent specificity of phage CBA120 for *E. coli* O157:H7 hosts. Likewise, binding to *E. coli* O157:H7 cells was not detected with fluorescently-labeled TSP1 by microscopy (data not shown). The results for binding to O157 LPS were mixed. TSP1 bound O157 LPS extracted from ATCC 43894 moderately (spot 5) but did not bind O157 LPS from a commercial vendor (spot 6). It remains to be determined whether these results represent heterogeneity in binding or are attributable to differences in extraction techniques and/or purity of the LPS. Nonetheless, with four TSPs, each with perhaps different binding epitopes and catalytic activities, the adsorption and infection process of CBA120 and other Viunalikevirus is likely more complex than contemporary phage.

### Is TSP1 an enzyme?

**Figure 7:**
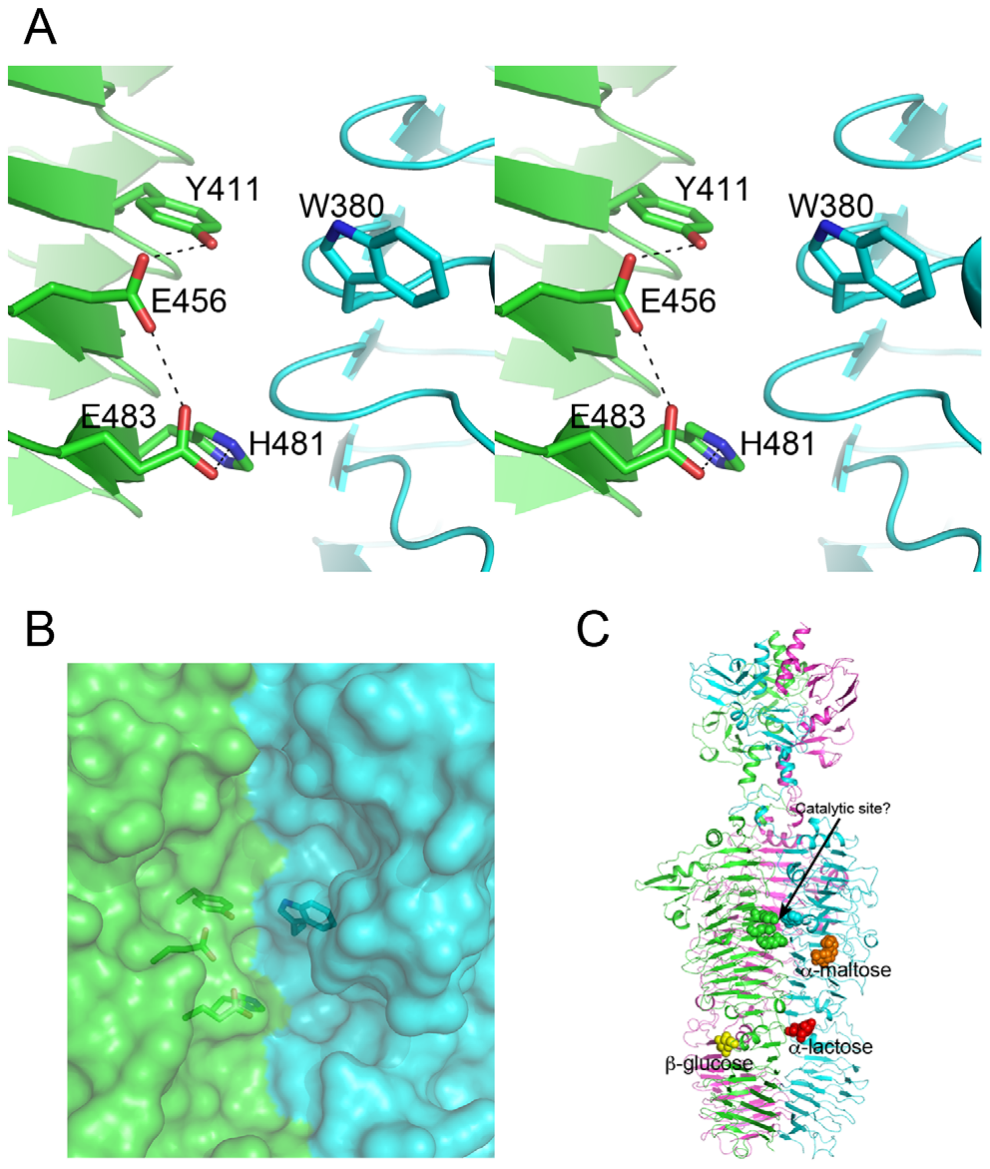
Potential TSP1 catalytic site (A) Stereoscopic representation of the potential catalytic center. The site is located in a groove at the subunit interface. The two subunits are colored green and cyan. (B) Surface representation showing the potential catalytic residues within a crevice that can accommodate substrate. (C) The catalytic center and the bound sugars within the context of the entire TSP1 structure.

Currently, there is a large number of TSP1 orthologs with homologous amino acid sequences spanning the head binding domain, but less than handful receptor binding domains. Multiple sequence alignment is therefore insufficient for locating the TSP1 active site based on sequence conservation pattern. Analysis of the structure of TSP1 shows that despite the broad fold similarity between TSP1 receptor-binding domain and those of other tailspikes receptor-binding domains with known active site residues, TSP1 does not contain any of the arrangements of tailspike catalytic residues reported in the literature. Instead, a cluster of amino acid residues located in a groove at the interface between subunits is suggestive of catalytic machinery (Figure 7). This cluster includes a pair of adjacent glutamic acids that share a proton, located on the same subunits of TSP1, Glu456 and Glu483 (Figure 7A). This is a recurring catalytic motif in the glycosyl hydrolases belonging to the chitinolytic enzymes of families 18, and 20 [36], [37], [38], and to the hyaluronidases of family 56 [39], [40]. His481 and Tyr411, both located on the same subunit, and Trp380 on the neighboring subunit may assist catalysis. The sugar-binding sites this study identified are located remotely from the proposed catalytic site (Figure 7C), suggesting that TSP1 acts on a glycosidic bond connecting different saccharide units. Identification of the true polysaccharide substrate will reveal whether any of the sugar binding sites observed in the three crystal structures reported here is utilized for binding the substrate.

The glycosyl hydrolase mechanism associated with two adjacent carboxylate groups that share a proton involves double displacement at C1 next to the glycosidic bond to be cleaved. The double displacement results in retention of configuration at C1. This mechanism has been proposed to be substrate-assisted whereby a substrate nucleophilic group, for example an N-acetyl group on the saccharide, provides an oxygen atom that acts as the nucleophile [38], [39]. The substrate-assisted mechanism has been identified in viruses belonging to the glycosyl hydrolase families 18 and 56 but not yet in tailspike proteins (see http://www.cazy.org/ for lists of family members). In contrast to TSP1, the Asp/Glu carboxylic groups of the defined tailspike catalytic machineries, whether located at a subunit interface (as in Sf2) or on a single subunit (as in P22, HK620, Det7), are placed to flank both sides of the glycosyl bond, ∼10 Å apart. Such carboxylate pairs operate by a single or two-step mechanism, the latter involving enzyme-substrate intermediate. If indeed the catalytic center of TSP1 utilizes the adjacent glutamic acid pair, Glu456/Glu483, the receptor may be a polysaccharide that contains a nucleophilic group such as N-acetyl. Studies to identify the TSP1 substrate, which in turn will facilitate site directed mutagenesis of the potential catalytic residues and structure determination of the protein/receptor complex, are in progress.
